# Comparative price analysis of biological medicines: disparities generated by different pricing policies

**DOI:** 10.3389/fphar.2023.1256542

**Published:** 2024-01-11

**Authors:** Marcela Amaral Pontes, Alane Andrelino Ribeiro, Flávia Caixeta Albuquerque, Silvana Nair Leite Cotenzini

**Affiliations:** ^1^ Pharmacy Department, University of Brasilia, Brasilia, Brazil; ^2^ Molecular Biology Department, University Catholic of Brasilia, Brasilia, Brazil

**Keywords:** biological medicines, biosimilars, drug price, regulation, access to health technologies

## Abstract

**Introduction:** Biological medicines have been assuming an important role among the therapeutic options for several diseases, however, due to their complex production process, the products obtained from this technology have a high added value and do not reach the purchasing power of most patients, which overwhelms the budget of health systems. With the development of biosimilars, which have reduced production costs, it is expected that access to biological medicines will become broader. However, in Brazil, the criteria for determining the price of biosimilars, unlike the generic policy in the country, do not foresee a price reduction due to the reduction of development costs.

**Objective:** To understand the impact of the current model of economic regulation on the availability and access of these products in the country, based on a comparative analysis in selected countries, and identify trends that can help to expand the availability and access to biological medicines.

**Method:** Quantitative and qualitative study, to identify the variation between the entry prices of biological medicines in Brazil and in selected countries, as well as the differences in the economic regulation policies established in these countries.

**Results:** The results demonstrate that the current pricing model in Brazil has generated distortions in the prices of biosimilars in the market, which, consequently, makes it difficult for the population to access this category of products, in addition to allowing unsustainable market practices for the systems of public and private health in Brazil. It was also found that most of the analyzed countries, unlike Brazil, seek to harmonize the prices of different brands of the same molecule marketed in the country and with the international market, in addition to establishing incentive policies for indication and replacement by biosimilars, which expands the participation of biosimilars in the market significantly.

**Conclusion:** Based on the data presented, it is concluded that it is essential to build a broader political and regulatory debate on the market for biologicals and biosimilars in the country to guarantee the access of the Brazilian population to more cost-effective technologies, generate a more competitive market and consequently contribute to the financial sustainability of health systems.

## Introduction

The growing number of biological medicines approved by regulatory agencies has generated the need for better understanding of the access to these technologies. However, the complex process of obtaining these products, the high investment in research and development, in addition to the market strategies, results in drugs with high added value, which do not reach the purchasing power of most patients and overload the budget of health systems (Bhatt, 2018; Sariahmed et al., 2022).

In Brazil, the National Health Surveillance Agency (Anvisa)–an agency linked to the Ministry of Health–is responsible for promoting the protection of the population’s health by overseeing the sanitary control of the production, commercialization, and use of products and services subject to sanitary regulations. The registration of biologic drugs began in 2002, based on specific rules that have undergone constant updates to align with international standards for the registration of pharmaceutical products. The first biosimilar registered in the country–infliximab–occurred in 2015. Currently, Brazil has around 500 registered biologics, including vaccines, blood-derived products, monoclonal antibodies, and advanced therapies (Brasil, 2010).

However, the diffusion of biological medicines is still comparatively lower than that of synthetic medicines due to factors such as high prices, limited number of diseases treated and the need for a developed health system to oversee treatments with this type of medicine. (Brasil, 2016; Brasil, 2018b; Brasil, 2023).

Treatments with biological agents are already quite significant for some therapeutic areas, especially in high-income countries. It is estimated that 19% of patients with rheumatoid arthritis in Europe had access to biologics in 2010. In 2014, 3.1 million patients in the US were treated with one of the seven best-selling and available biologics in the country (Sengupta, 2018).

The World Health Organization (WHO) has been including new biological medicines in each edition of the list of essential medicines. In 2015, trastuzumab and rituximab were included, and in 2019, adalimumab and nivolumab. Previous lists had already included bevacizumab, pegylated interferon alpha and filgrastim (WHO, 2021b).

According to data released by the Chamber of Regulation of the Pharmaceutical Market (CMED), the sales of biologic medicines in Brazil in 2022 represented 26% of the total revenue of pharmaceutical companies and only 1.6% of units sold. Among the top 10 therapeutic classes by revenue, four are related to biologic products: coronavirus vaccines, anti-TNF (tumor necrosis factor) products, monoclonal antibodies for oncology (PD-1/PD-L1), and HER-2. According to Mega (2019), 40% of the federal public budget for pharmaceutical assistance is used to acquire biologic medicines, which serve around 2% of the total patients treated in the Brazilian Unified Health System (SUS).

The Organization for Economic Cooperation and Development (OECD) and the WHO have warned of the increased availability of high-priced medicines and questioned the current pricing models for these products in the world, since it is clear that high prices can make these medicines inaccessible, compromising equitable access and threatening the financial sustainability of health systems (WHO, 2011; OECD, 2018).

The development of biosimilars, defined as biological medicines that have a high similarity in quality, efficacy, and safety with the approved originating biological medicine, was carried out with the aim of reducing the production costs of biologicals with an expired patent. According to data from IQVIA (2020), the costs of biosimilars in Europe are about one-third of the originator biologicals. List prices are highly variable and depend on the health system and product model. It is also noted that, in addition to the reduced cost, the confidential discounts applied in the price negotiation process vary between 10% and 90%.

For biosimilar medicines to become the ideal way to expand access to biotechnological treatments in Brazil, there is a need for public and private investment in innovation, research, and development of biopharmaceuticals, with the objectives of increased competition in the Brazilian market and lower import dependency. It is also necessary that the sanitary and economic regulations of the pharmaceutical market understand the differences involved in the production process of this category of products and establish rules that help in the access to effective and safe products, with prices that reflect the reduction of research, development, and production costs, foreseen in production processes of similar products with expired patent.

The CMED, the body responsible for establishing criteria for setting and adjusting drug prices, published Communication No. 9 in 2016, containing rules for pricing “non-new biologics.” This regulation foresees the use of methodologies such as external or internal referencing to determine the price of biologic medicines. The term “non-new biologics” began to be used by CMED to classify biologic products developed through individual development or comparability pathways, also known as “biosimilars” in various countries.

The pricing methodologies practiced by CMED are widely used in countries with price regulation policies. However, when it comes to setting prices for biosimilar drugs, it is observed that many European countries use the price link methodology, which involves fixing a percentage discount on the price of the reference or originator drug to determine the price of a generic or biosimilar medicine (Vogler et al., 2021). This discount on the price of the originator biologic medicine ranges from 15% to 30%, depending on the country (Vogler et al., 2021). In Pakistan, it was identified that the price of the first biosimilar can be reduced by up to 30% compared to the reference medicine (Babar, 2022).

Based on the highlighted points, this study intends to analyze the evolution of the entry price of biological and biosimilar medicines in Brazil over the years and establish a parallel with the pricing policies of this class of medicines in the countries used as an external price reference by Brazil, with a view to identify how the current pricing model behaves in the Brazilian market and what is its impact on access and availability of these products in the country.

## Methodology

Based on data from the “Statistical Yearbook of the Pharmaceutical Market”, 2019/20 edition (Brasil, 2021a), seven biological medicines were selected among the 20 substances with the highest revenues in the country in 2019. For each active ingredient selected, concentrations and pharmaceutical forms available in the Brazilian market and in the countries defined in CMED Resolution 2/2004 (Brasil, 2004) as an external reference price (ERP) were identified, which generated a list of 11 different presentations.

After defining the presentations, the Ex-Factory Prices (FP) were collected, that is, without taxes, in all price lists published and available on the official websites of the selected countries, as presented in Table 1. This search generated data from 2003 to 2022, depending on the country and medicine. Data collection took place between August 2021 and January 2023.

**TABLE 1:** Price research websites by selected country.

Country	Price research website
Brazil	www.gov.br/anvisa/pt-br/assuntos/medicamentos/cmed/precos
Australia	www.pbs.gov.au/pbs/industry/pricing/ex-manufacturer-price
New Zealand	www.schedule.pharmac.govt.nz/ScheduleReporting.php
Canada	www.idbl.ab.bluecross.ca/idbl/load.do
	www.ramq.gouv.qc.ca/en/about-us/list-medications
United States	www.department.va.gov/administrations-and-offices/acquisition-logistics-and-construction/freedom-of-information-act-requests/
Spain	www.sanidad.gob.es/profesionales/farmacia/financiacion/home.htm
France	www.codage.ext.cnamts.fr/
Greece	www.moh.gov.gr/articles/times-farmakwn/deltia-timwn
Italy	www.aifa.gov.it/web/guest/liste-farmaci-ah
Portugal	www.infarmed.pt/web/infarmed/servicos-on-line/pesquisa-do-medicamento

The database built in Microsoft Excel includes the active principle, concentration, pharmaceutical form, regulatory category (originating biological or biosimilar), brand name, quantity of pharmacotechnical unit per packaging, year, and FP for each year.

With the database built, the prices of drugs that have a patent in force and drugs that already have biosimilars on the market were compared, separately, in order to understand the different methodologies applied by the selected countries in the definition of the entry price of the different regulatory categories of biopharmaceuticals (biologics and biosimilars). For comparison purposes, drug prices were calculated per presentation and converted according to each country’s purchasing power parity (PPP). PPP is an alternative method to the exchange rate, widely used for international comparisons and measures how much a particular currency could buy if it were not influenced by the market or economic policy reasons that determine the exchange rate. The calculation of the PPP is carried out and released by the World Bank and is based on the US dollar. For conversion purposes, the 2022 PPP was used in this study (OECD, 2023).

The prices collected were not adjusted for inflation since the prices displayed in the public lists, per year, are adjusted according to inflation or other adjustment methodologies, according to the country’s economic regulation rules.

In addition, a documentary survey of normative acts and legislation in force was carried out to identify historical and conceptual elements related to the regulation of prices of biological medicines in the selected countries. The documentary research took place on the websites of organizations and government entities, such as health regulatory agencies, health technology assessment agencies and ministries of health.

## Results

The seven biological medicines objects of this analysis, their respective presentations, and brands, as well as the prices registered in the selected countries in 2022, adjusted by the 2022 PPP and exempt from taxation, are presented in Table 2, where it is possible to identify that the prices of the biological medicines in Brazil are among the highest compared to the selected countries. It is noted that there is a considerably large difference in price variations between Brazil and selected countries for originator biological medicines that have biosimilars in the market and for biological medicines with a valid patent. Remicade FP in Brazil in 2022 was 1,054% higher than in France. The variation in prices of medicines with a valid patent is much smaller, for example, FP of Perjeta in Brazil is 159% higher than in Italy. In the comparative analysis of prices adjusted by the 2022 PPP, exempt from taxes and fees, it is reaffirmed that the entry price in Brazil, after years of commercialization, is the highest among the referenced countries, approaching only the United States. The cells without data in Table 2 may be related to the non-commercialization of the product in the market or the absence of a price in the official lists of the countries surveyed.

**TABLE 2:** Comparison of FP in PPP dollar in selected countries in 2022.

Medicine (mg)	Brand	BRA (US$ PPP)	AUS (US$ PPP)	NZL (US$ PPP)	CAN (US$ PPP)	US (US$)	ESP (US$ PPP)	FRA (US$ PPP)	GRE (US$ PPP)	ITA (US$ PPP)	POR (US$ PPP)	Variation (%)between BRA price and lowest price
Bevacizumab 100	Avastim	675.37				765.78	**105.42**	107.73	179.22			541
	Mvasi		84.37			687.22	163.48	103.58	143.38			
	Zirabev				108.85	526.29	114.43	90.93	143.38			
	Alymsys					707.77		103.58	143.38			
	Oyavas							103.87	143.38			
	Aybintio								143.38			
Bevacizumab 400	Avastim	2,614.96				3,063.12	**421.10**		717.77			521
	Mvasi		337.48			2,748.81			574.21			
	Zirabev				435.41	2,105.17	421.10		574.21			
	Alymsys					2,831.07			574.21			
	Oyavas								574.21			
	Aybintio								574.21			
Infliximab 100	Remicade	1,613.89	221.79	749.60	**139.85**	610.41	140.94			558.95	253.79	1.05
	Remsima	919.40			**139.85**				238.33		215.89	557
	Biomanguinhos	1,589.32										
	Renflexis	610.36	**221.79**	393.14		504.58						175
	Inflectra		221.79				91.61		238.33			
	Flixabi								238.33			
	Xylfia	1,565.06		418.66							**200.30**	274
	Avsola			393.14								
	Zessly								238.33			
Nivolumab 100	Opdivo	3,242.15	1,364.39				**701.70**		829.25	1,823.83		362
Nivolumab 40	Opdivo	1,296.86	545.76		339.72		**280.68**		331.95	729.53		362
Pembrolizumab 100	Keytruda	5,835.88	2,644.36		2,125.34		**1,796.67**		2,115.08	3,245.49		225
Trastuzumab 150	Herceptin	1,665.15			237.48		**169.54**		356.61	936.20		882
	Ogivri		163.91				169.54					
	Trasimera	739.45	**163.91**				169.54					351
	Kanjinti	739.45	**163.91**				169.54		285.28			351
	Herzuma	877.96	**163.91**		237.48		169.54		285.28			436
	Ontruzant	847.16	**163.91**				169.54		285.28			417
Trastuzumab 440	Herceptin	4,151.32					**474.72**		1,019.89	2,687.24		774
	Ogivri						474.72		798.79			
	Zedora	4,884.16							**798.79**			511
	Trasimera	2,169.05					**474.72**		798.79			357
	Herzuma	2,575.34	**480.82**						798.79			436
	An intruder	2,485.01					**474.72**		798.79			423
	Kanjinti	2,114.43	**458.96**				474.72		798.79			361
Rituximab 100	Mabthera	1,322.29								**745.84**		77
	Rixymio	1,322.29	234.99							**190.94**		593
	Tricks	1,322.29					**97.85**					1.25
Rituximab 500	Tricks	3,300.73	**234.99**				489.24					557
Pertuzumab 420	Life	4,381.05	2,018.50				1,784.53		**1,691.59**	2,723.30		159

The highest and lowest prices of the brands available in Brazil and other countries are highlighted in bold.

Table 3 details the pricing and price review rules in the selected countries and demonstrates that European countries and Australia have policies for reviewing and/or reducing the prices of biological medicines with or without a valid patent. These countries tend to harmonize the prices of different brands of the same molecule marketed in the country with the international market, based on the price link methodology, defined as the establishment of a percentage discount on the price of the reference or originator medicine to determine the price of a generic or biosimilar medicine (Vogler et al., 2019). In Greece, prices are revised annually based on the average of the 2 lowest prices in the European Union and cannot be reduced by more than 7% of the current list price. In France, prices are revised after 3 or 5 years, according to the evaluation of the therapeutic progress of the medicine, and with the entry of biosimilars into the market, so that the prices of the active ingredient under analysis are harmonized, regardless of whether it is the originator biologic or biosimilar. New Zealand, due to the pricing model, which is linked to the process of purchasing medicines for the public health system, does not revise prices periodically. Brazil and the United States also do not have established criteria for price revision.

**TABLE 3:** Biosimilars pricing policy and biologicals and biosimilars pricing review.

	Pricing methodology	Price review
BR	External price referencing (REP) for biosimilars that demonstrate clinical benefit.	There is no price revision rule
	Internal price referencing (RIP) for biosimilars already on the market.	
AU	The reduction of the new brands will be based on previous price reductions. For example, if the first brand has reduced by 35% or less in 2016, the price of the new brand should not exceed the PF of the existing brand reduced by 25%.	5% reduction after 5 and 10 years on PBS. 26.1% or 30% after 15 years.
NZ	There is no specific rule set	There is no price revision rule
CA	There is no specific rule set	The revision of patent medicines prices considers an adjustment factor based on inflation and should not exceed the highest price among the comparison countries.
US	Does not have a drug price regulation policy	Does not have a drug price regulation policy
ES	Price link: −30% from originator	Annual review according to the sales and commercialization of new drugs of the same therapeutic class.
FR	Price link: −40% from originator and reduces originator 20%. After 18 and 24 months, further reductions (5%–15%) occur according to market share. Hospital: −30% biosimilar and originator	Review after 5 years of marketing for drugs with ASMR I to III and for other cases after 3 years. After 1 year of commercialization of the biosimilar, the price of the originator medicine can be revised to harmonize prices.
GR	Expired patent: −20%	Annual review and follows the same entry price definition rule (average of the 2 lowest prices in the EU).
	Biosimilar medicines: average of the 2 lowest prices in the EU.	
IT	Price link: −20% from the originator	Review from 36 months for innovative drugs and 18 months for drugs with potential innovation. May occur due to new therapeutic indication, dosage, or scientific evidence.
PT	Reimbursed medications: −20% or −30% for BP with a market share greater than 5%.	Annual review based on the REP or extraordinary according to the justification presented to INFARMED.

1CMED Communiqué 9, of 10 August 2016; 2National Health Act 1953; 3Clarivate Analytics. Cortellis for Regulatory Intelligence. Regulatory Summary Expert–Pricing and Reimbursement (New Zealand). 2021; 4Compendium of Policies, Guidelines and Procedures 2022; 5Vogler et al, 2021; 6Royal Legislative Decree 1/2015, of July 24; 7Accord-cadre du 03/05/2021; 8PPRI, Pharma Brief: Greece 2007; 9PPRI, Pharma Brief: Italy 2021; 10 Decree-Law 97, of 1 June 2015. AMSR: Amélioration du service médical rendu (improvement in medical benefit). INFARMED: national authority for medicines and health products.

In the analysis of the historical prices of biologicals with a valid patent, it is observed that nivolumab, after 5 years of commercialization in Brazil, had its price adjusted by 30%. Pertuzumab, with 8 years on the market, increased the FP by 35%, and pembrolizumab, in 4 years, had an increase of 28% over the FP. This percentage increase is even higher than in the United States, a country known for charging the highest prices for most medicines in the world (Daalen et al., 2021).

**FIGURE 1:**
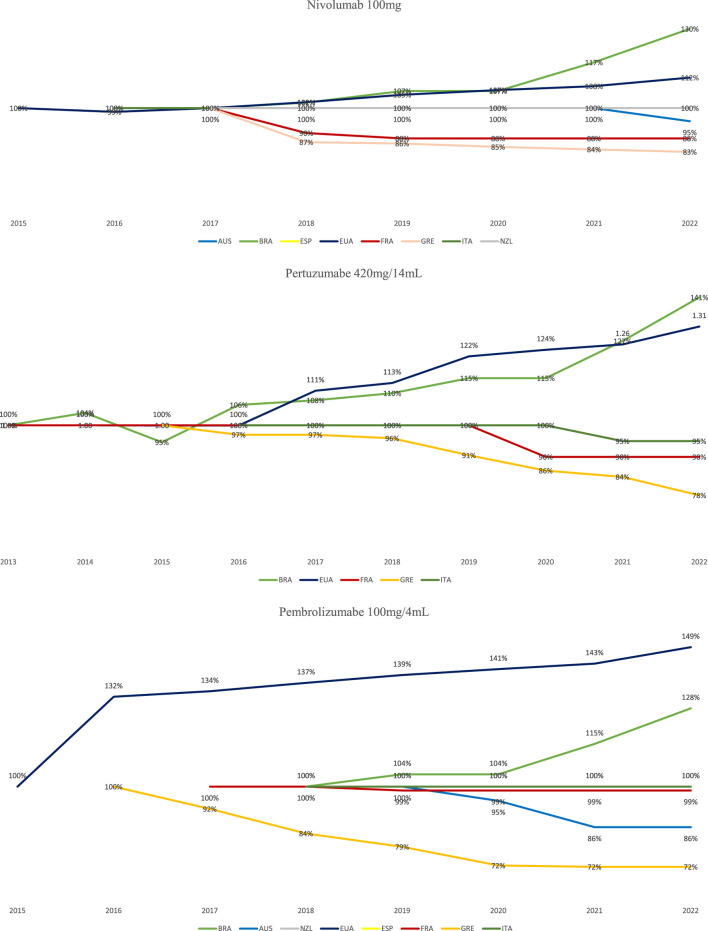
Prices course of biological medicines with a valid patent in selected countries, from 2013 to 2022.

European countries and Australia register the biggest discounts in the entry prices of medicines with valid patent. Greece, through its annual review policy, has the greatest reduction in prices, for example, the price of nivolumab, after 5 years on the market, has reduced by 17%. Pertuzumab reduced by 22% after 7 years in the market and pembrolizumab had its price reduced by 72% after 6 years of introduction into the country (Figure 1).

**FIGURE 2:**
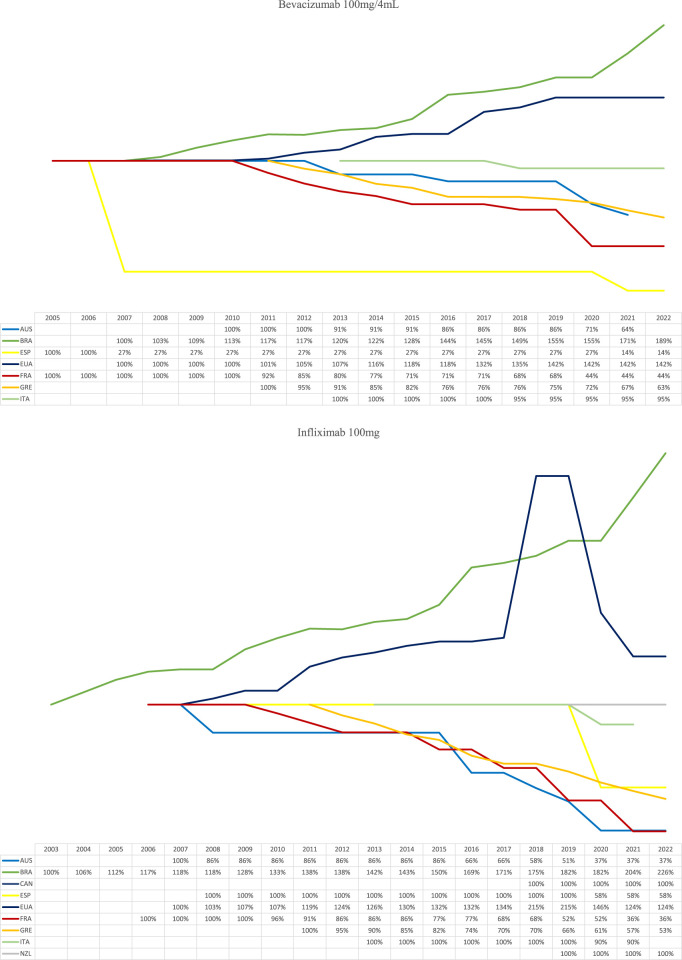
Price behaviour of biological medicines with expired patent in selected countries, from 2013 to 2022.

Figure 2 shows the price course of medicines with expired patents, that also have biosimilars on the market. Australia and European countries drastically reduce prices with the entry of biosimilars. As shown in Table 2, countries such as Australia, Spain, France, and Portugal use the price link methodology, while Greece and France also apply a reduction rate in the originator biological price to harmonize the prices of different brands of the same molecule. In Brazil, even with the entry of biosimilars into the market, the price of the originator biologic has been constantly growing.

**FIGURE 3:**
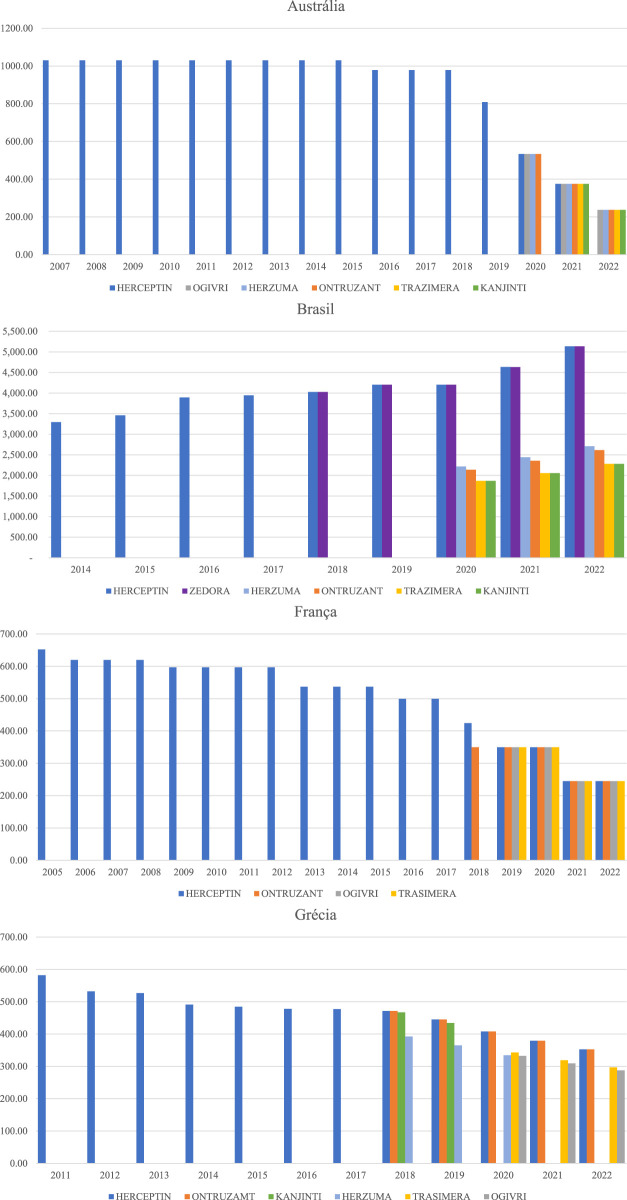
Trastuzumab 150 mg originator biological drug price history and biosimilars.

**FIGURE 4:**
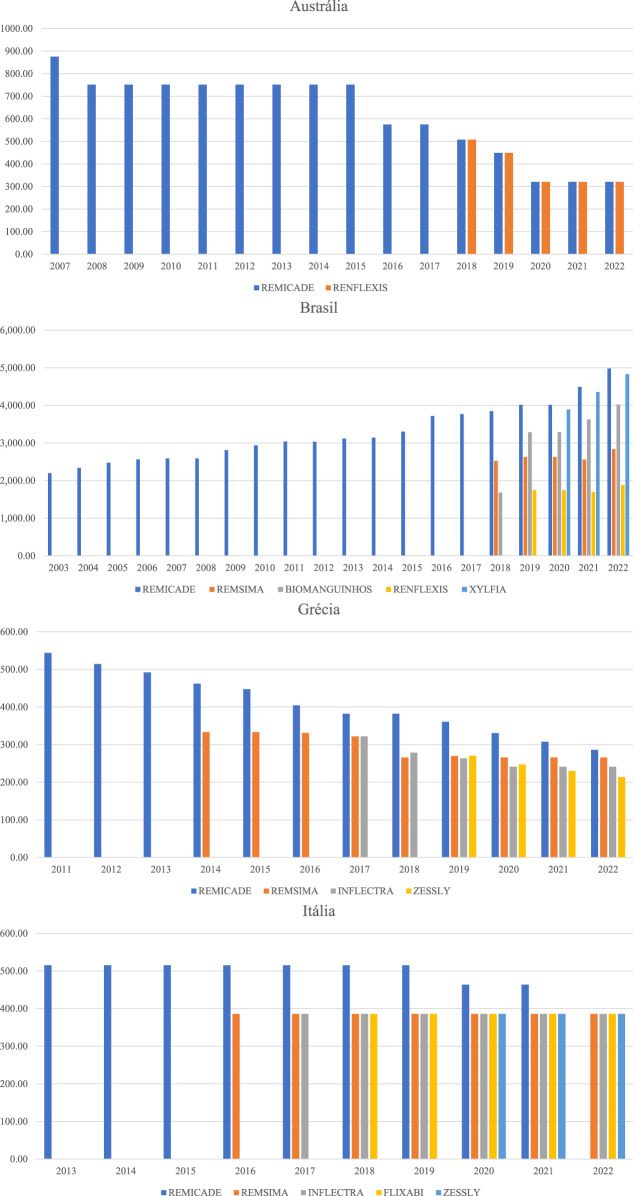
Price history of the originating biological medicine and biosimilars of Infliximab 100 mg.

Figures 3, 4 detail by country how the list prices of the originator biologics and biosimilars behave with the entry of new brands into the market. The values in the Figures are presented in the currency of the country analyzed. Infliximab 100 mg and Trastuzumab 150 mg were used as examples because they have a greater number of biosimilars on the market in the countries studied.

Hen observing the evolution of the prices of biological originators of trastuzumab 150 mg (Herceptin) and infliximab 100 mg (Remicade), it is noticed that Brazil generates great distortion in the prices of similar presentations. FP of Trastuzumab in 2022 ranged from BRL 2,281.46 to BRL 5,137.60, which is equivalent of a difference of 125% between the lowest and the highest price. The FP of infliximab had a variation of 164%. Greece, due to the annual price review, manages to generate a much lower variation between the prices of biologicals with a similar molecule than in Brazil, that is, the prices of trastuzumab available in the Greek market varied 23% in 2022, and the prices of infliximab, 34%.

Australia, France, and Italy define rules for economic regulation that establish a percentage for reducing the price of biosimilars and, by establishing periodic price reviews, guarantee the same FP for different brands of the same molecule, which generates better competition with the potential to expand access to medicines. The entry price of the infliximab biosimilar in the Australian market was 42% lower than the originator entry price. In France, the trastuzumab biosimilar had its entry price recorded at 54% of the originator’s value.

## Discussion

This study presents the evidence for biological medicines price variation in Brazil and compares it with the prices in the countries used as an ERP for defining the entry price. The study identifies that the Brazilian population has access to biological medicines with some of the highest prices among the countries compared. This study corroborates the findings of Analytics (2021) and Moye Holz and Vogler, (2022), who identified that high prices are one of the causes of lower access to biological medicines for Latin American citizens.

The current methodology used in Brazil for pricing originator biologicals and biosimilars is based on ERP or IRP, by calculating the cost of treatment with therapeutically comparable drugs. These rules, according to data presented, have generated significant distortions in prices and do not help in the development of a market with perfect competition.

According to Holtorf et al. (2019), several authors have already concluded that ERP causes some reduction in drug prices, but there is little evidence on the concrete impact of this methodology on price, access, availability, quality, and the health system in the long term. This study demonstrated that the ERP has been described as an inefficient approach to reducing prices when used in isolation from other methodologies and, therefore, more value is seen when there are combinations of pricing policies. Another questioning that has been carried out in several discussion spaces about the use of the ERP is related to the selection of reference countries, which should consider nations with similar geographical proximity, income, availability of medicines, and market size, to guarantee that the definition of the price is adequate to the socioeconomic condition of the country (WHO, 2021a).

One of the objectives of using the ERP is to try to ensure that the price paid for a pharmaceutical product in Brazil does not excessively exceed the price paid in the countries it is compared to. However, other characteristics of the Brazilian model distance and distort these prices in the market. The high tax burden, the US dollar exchange variation, inflation and the lack of periodic monitoring and revision of prices make the availability of products in the market and access to medicines in Brazil increasingly difficult. In 2012, the report on judgment 3016 of the Federal Court of Auditors had already recommended that the Ministry of Health review and correct the regulatory model provided for in Law 10,742/2003, to detach inflation adjustments, as they found that 86% of drugs from a sample of drugs with the highest revenues were priced above the international average, with 46% having the highest price in Brazil (Brasil, 2012; Brasil, 2003a; Brasil, 2003b; Dias et al., 2019).

The Federal Court of Auditors also highlights the need to adapt the current economic regulation policy to make it more flexible and establish rules for reviewing prices in the country. In this context, WHO recommends providing information on rebates, discounts or other transactions between sellers, sponsors, and payers/buyers (WHO, 2019). The opacity of this information is an important component in the financial unsustainability of access to medicines by citizens and other payers, and transparency and information sharing has the potential to provide evidence for decision makers, to guide more accessible prices (Ribeiro et al., 2023a; Ribeiro et al., 2023b).

Price link methodology for defining biosimilar prices can be an alternative to generate a market in which competitors operate under similar conditions. In most of the analyzed European countries, it was observed that this has been a more efficient policy to align the prices of products with the same or similar therapeutic effects and to reduce price variability between comparable products.

In Brazil, the price link methodology is only used to define the prices of generic drugs, which must have their prices published with up to 35% discount on the price of the reference drug. For biosimilars, in addition to the need to improve the pricing policy, there is a lack of definition of the concept of this type of medication and the creation of a policy to encourage the use and replacement of these products, as occurred with generics with the publication of the Generic Law in 1999 (Brasil, 2019). These flaws in the execution of public policy generate price distortions in the market, do not stimulate the prescription and use of biosimilar products and create more barriers for a more competitive market. Countries in Europe that have incentives aimed at prescribers for the indication and replacement of biosimilars generated, for example, a market share of more than 95% for the biosimilar Infliximab and the increase to more than 82% of market share for the biosimilar Etanercept (Moorkens et al., 2021; Vogler et al., 2021).

In Ireland, due to low uptake of the use of biosimilars and the increasing availability of these products in the market, led the Health Service Medicines Management Executive Programme (HSE-MMP) to publish a guide to the prescription of value-based biologics in December 2018. This guide defines criteria for choosing biosimilars that will be used in the health system, based on the cost of acquiring the drug, therapeutic indications, range of products available, product stability, delivery devices, clinical guidelines, capacity to supply the Irish market and the potential savings. By applying the criteria set out in the guide, savings of €22.7 million were estimated by June 2020 (Duggan et al., 2021).

Kim et al. (2020), based on the sales values of biologicals in the United Kingdom, France, Japan, and South Korea, showed that the entry of the biosimilar infliximab decreased the market share of the originator in the United Kingdom, France, and Japan, in addition to confirming the price reduction of biosimilars in relation to the originator. One of the causes for this result is due to government actions aimed to increase the penetration of biosimilars in the market, as is the case in the United Kingdom and France, a country that has a defined interchangeability policy. In South Korea, the entry of biosimilars generated a phenomenon contrary to the other countries analyzed, that is, there was an increase in the use of the originator and the biosimilar, and the author attributes this situation to the deficiency of specific policies for the use of these products in the country.

Carl et al. (2022) compared prices of biosimilars in the US, Germany, and Switzerland over the period 2011 to 2020 and found that prices of biosimilars and originator biologics were substantially higher in the United States compared to Germany and Switzerland. A possible reason for the limited availability of biosimilars in the United States could be an ongoing patent litigation or agreements to defer entry as a result of patent dispute resolution. He also highlighted that the limited availability of biosimilars in the United States may be a result of scepticism among prescribers and patients regarding the efficacy and safety of biosimilars. Biosimilar prices compared to originators ranged more widely in the United States (between 55% and 90%) and Germany (between 65% and 103%) compared to Switzerland (between 70% and 80%). The results for Switzerland can be explained with the price link policy. On the other hand, Germany does not consider the prices of the originator biologicals when negotiating the prices of biosimilars, which can lead to prices of biosimilars being higher than those of originators.

In 2018, Brazil created a working group to discuss and formulate the National Policy on Biological Medicines in the Unified Health System (SUS). Among the guidelines elaborated, the priority is the development of normative acts related to the interchangeability of biological medicines, based on the best available scientific evidence, to prevail the user safety, the public interest, and the expansion of access (Brasil, 2018a). The group held several discussions and propositions that so far have not been put into practice. However, biological products represent about 60% of public spending on medicines in Brazil, despite involving only 12% of the quantity of medicines, indicating urgent intervention to regulate this market (Brasil, 2018b).

The importance of including biosimilars in public health is strongly related to the costs of biological originators and the demographic and epidemiological profile of the population, therefore, the adoption of policies to encourage the use of these products can lead to considerable cost savings for the population and for systems health, in addition to expanding access to new technologies (Mosegui et al., 2021).

In addition, Brazil annually performs a positive price adjustment according to inflation and sector costs, without establishing any realignment of entry prices. According to the price cap model of economic regulation, the regulator must define the maximum amount to be charged for products/services and assumes periodic realignment of prices to market values, in accordance with efficiency gains and changes in the regulatory scenario. The usual review period is between 3 and 5 years, and, annually, the values can be readjusted by some inflation index (Brasil, 2012).

The Administrative Council for Economic Defense points to the use of inappropriate practices in the acquisition of biologicals in the private market due to the current distortion in the entry prices of biologicals. The CMED list is used by health insurance companies as a reference value for reimbursing hospitals, which results in choosing to buy the most expensive biologics and rely on their negotiating power to guarantee significant price discounts and generate a greater reimbursement margin for hospitals (Brasil, 2021b).

Another ineffective practice that stands out in the market for biologics, and for high-cost drugs, is the negotiation of prices during the process of incorporating technologies into the SUS. The National Commission for the Incorporation of Technologies in the SUS (Conitec) uses, as a basis for price negotiation, entry prices published monthly by CMED, and public purchases made available in the Health Price Database (BPS). However, at the time of acquisition, the recommended prices for incorporation into the SUS are not necessarily used as a basis for purchase by subnational public institutions.

Among the drugs analyzed in this study, it was observed that the initial price proposal by the pharmaceutical company for the incorporation of Pertuzumab, and purchase by SUS in 2018, was R$ 4,199.34 (FP0%), that is, a 50% discount on the price of the CMED list. However, in 2022, according to data published in the BPS, state purchases were made with a Maximum Sale Price to the Government (PMVG 18%) of R$ 10,479.08, (Brasil, 2019). Assuming that the price suggested by the pharmaceutical company in 2018 was adjusted in 2022, according to the cumulative adjustment for the period from 2018 to 2022, that is, 30.20%, it can be seen that the prices of state purchases occurred with prices much higher than those initially suggested for incorporation into the SUS. However, this price is within the PMVG published in the 2022 CMED list, that is, R$ 10,606.89.

When it comes to centralized purchasing by the federal government, Mega (2019) observed that unit prices between 2012 and 2017 reduced, on average, by 28% for 10 biologics analyzed. However, some products showed drops of more than 40%, such as Abatacept 250 mg (49%), Tocilizumab 20 mg (46%) and Golimumab 50 mg (40%) and Abatacept 125 mg (155%). In the same period, the CMED allowed a cumulative annul adjustment of the FP by 23.91%. Mosegui et al. (2021) identified that federal purchases of oncological biologics, carried out between 2015 and 2019, did not generate savings in resources when opting for the purchase of biosimilars. The influence of biosimilars on the prices of reference biologics was not evident.

These data point to some reasons that lead to price variation in public procurement, such as the presence or absence of competition in the market, the negotiation capacity and purchasing power of the federative entity, or even the availability of the product from national production, and the lack of a well-established policy to encourage biosimilars. The above-mentioned results also demonstrate that the negotiations carried out during the process of incorporation into the SUS and the process of public procurement do not guarantee that the health system will be able to acquire medicines with significant discounts on the FP, since the current legislation determines that any acquisition must consider the list price of the CMED as the maximum price, which has been shown to be much higher than the actual prices.

With the evidence presented here, the need for a broader political and regulatory debate on the biologics and biosimilars market in the country is reinforced, to guarantee the access of the Brazilian population to more cost-effective technologies, generate a more competitive market and consequently contribute for the financial sustainability of health systems.

This study has some limitations, such as a small sample of biologicals that does not allow extrapolating the results to the entire market. It is not possible to conclude that the price reductions of biologics and biosimilars in the countries analyzed are the real prices practiced, because some countries use a regulatory methodology complementary to the REP and the price link–price negotiation–which is confidential and, therefore, it may be that the prices of biologics have different percentage variations from those presented. In addition, the countries analyzed have different health systems with different economic regulation policies.

## Data Availability

The original contributions presented in the study are included in the article/supplementary material, further inquiries can be directed to the corresponding author.
